# 'You have to put a lot of trust in me': autonomy, trust, and trustworthiness in the context of mobile apps for mental health

**DOI:** 10.1007/s11019-023-10146-y

**Published:** 2023-03-30

**Authors:** Regina Müller, Nadia Primc, Eva Kuhn

**Affiliations:** 1 Institute of Philosophy, University of Bremen, Enrique-Schmidt-Str. 7, 28359 Bremen, Germany; 2 Institute of History and Ethics of Medicine, Heidelberg University, Im Neuenheimer Feld 327, 69120 Heidelberg, Germany; 3 Section Global Health, Institute of Hygiene and Public Health, University Hospital Bonn, Venusberg-Campus 1, Bonn, Germany

**Keywords:** Digitization, mHealth, O'Neill, Obligation, Avatar, Bioethics

## Abstract

Trust and trustworthiness are essential for good healthcare, especially in mental healthcare. New technologies, such as mobile health apps, can affect trust relationships. In mental health, some apps need the trust of their users for therapeutic efficacy and explicitly ask for it, for example, through an avatar. Suppose an artificial character in an app delivers healthcare. In that case, the following questions arise: Whom does the user direct their trust to? Whether and when can an avatar be considered trustworthy? Our study aims to analyze different dimensions of trustworthiness in the context of mobile health app use. We integrate O'Neill's account of autonomy, trust, and trustworthiness into a model of trustworthiness as a relational concept with four relata: B is trustworthy with respect to A regarding the performance of Z because of C. Together with O'Neill's criteria of trustworthiness (honesty, competence, and reliability), this four-sided model is used to analyze different dimensions of trustworthiness in an exemplary case of mobile health app use. Our example focuses on an app that uses an avatar and is intended to treat sleep difficulties. The conceptual analysis shows that interpreting trust and trustworthiness in health app use is multi-layered and involves a net of interwoven universal obligations. At the same time, O'Neill's approach to autonomy, trust, and trustworthiness offers a normative account to structure and analyze these complex relations of trust and trustworthiness using mobile health apps.

## Introduction

Kyle has had problems falling asleep and maintaining sleep for quite some time. During a regular visit at their^1^ General Practitioner (GP) Kyle mentions their sleeping troubles to their physician. Their GP assumes that Kyle might have mild insomnia or at least sleeping difficulties that need some action to avoid chronification or worsening of their sleep disturbances. The GP prescribes an application (app) for the treatment of sleep disorders that is based on cognitive behavioral therapy (CBT)^2^ and that Kyle can download from an app store and use at home on their own (a so-called stand-alone app). After the download, Alex, an avatar, introduces himself on the screen as Kyle's personal sleep coach. The avatar asks Kyle their name and explains that he will guide Kyle through the different modules of the mobile sleep app. At the start of the structured program, the avatar asks Kyle about their current sleep habits and problems. At the end of this first assessment, the avatar says: 'I am well aware that you have to put a lot of trust in me on this journey. Maybe you're wondering why, of all people, you should trust me. So, I would like to promise you something. [Kyle], I promise you that all the techniques, exercises, and advice I'll give you are scientifically thoroughly researched and effective methods. Thirty years of research from us sleep experts prove that we can significantly improve your sleep with our methods. I am sure that you will benefit from this training in the long term' (Somnio 2022). In this sequence which is directly quoted from the health app somnio^3^ Alex, the avatar, thus, asks for Kyle's trust and presents himself as 'trustworthy'.

### Trust, trustworthiness, and mHealth

Trust and trustworthiness are essential for good healthcare (Dawson 2016). Especially in mental healthcare, trust plays a key role and has multiple facets, ranging from public trust in health services to trust between the individual service user and provider. New technologies such as mobile health (mHealth) apps, mainly when based on artificial intelligence (AI), can affect trust relationships between patients or users and physicians (Nundy et al. 2019). The impact may manifest at different levels. If AI-based systems are used only to supplement physician's expertise, the impact of AI on the trustworthiness of clinical encounters may prove minimal.

In contrast, if AI-based systems are intended to replace human clinical expertise, the impact on the relationship is more difficult to predict (Mittelstadt 2021). For example, the development of trust in a patient-physician relationship may be hindered by technological mediation. As mediators, AI-based systems can lead both the physician and the patient to talk about health exclusively in terms machines can measure or interpret. Technologies that prevent the communication of psychological signals and emotions can undermine the establishment of a trusting and healing patient-physician relationship (Mittelstadt 2021). These possibilities suggest that the encounters in which the basic trust develops (which is necessary for a patient-physician relationship) may be compromised by technological mediation (Mittelstadt 2021).

If a human physician or psychotherapist does not deliver healthcare, but a mHealth app does, one may wonder to whom Kyle's trust is directed. Although the avatar Alex explains why Kyle should trust *him*, one could argue that it is instead the technology or the company behind the mHealth app that Kyle is asked to trust. Besides, trust may be related to various aspects of the app, such as privacy, effectiveness, or quality.

Applied ethics and philosophy of technology, have taken up 'trust' as a key ethical concept in debates on the use of digital and mobile technologies, often focusing on AI-based technologies. While some authors argue that AI cannot be a target of trust (e.g., Hatherley 2020; Ryan 2020), others discuss trust in non-human agents (Ferrario et al. 2021) and technology concerning its technical, interpersonal, and institutional dimensions (Weydner-Volkmann and Feiten 2021). In the healthcare context, some follow an account of trust as a relationship between AI applications, AI practitioners (users or patients), and healthcare professionals (Nickel 2022). In contrast, the practice-oriented approach of the European Commission's 'Ethics Guidelines for Trustworthy AI' considers lawfulness, ethicalness, and robustness as the three components of trustworthy AI (European Commission's High-level Expert Group on AI 2019). Most current discussions on trust ignite at the use of technology that is based on AI. The arguments brought forward in this debate can largely be transferred to mHealth technologies in general. First accounts to capture the trustworthiness of mHealth apps have been made, for example, by presenting trustworthiness checklists for mHealth apps (van Haasteren et al. 2019, 2020). Beyond that, qualitative studies on mHealth apps such as sleep tracking devices, revealed that trust is a relevant issue for the users of these apps (e.g., Liu et al. 2015). Hence, it has been concluded that health apps 'must also engender trust' if they ought to have therapeutic value (Torous and Roberts 2017, p. 438).

### Objective of the present study

As this brief overview shows, trust and trustworthiness are discussed as essential aspects in the use and successful implementation of mHealth. However, there is still a lack of an encompassing account that allows categorizing the various aspects of trust in mHealth mentioned in our introductory example. As will be argued, O'Neill's philosophical account of trust and trustworthiness, which has been developed for bioethics, offers such an encompassing philosophical account (O'Neill 2002, 2018). In the following, we first give an overview of the technological background and issues of trust and trustworthiness in the context of mHealth, focussing on stand-alone apps for treating sleep difficulties with CBT. Second, we introduce O'Neill's account of autonomy, trust, and trustworthiness. As O'Neill's account has not been explicitly developed for the field of mHealth and, to the best of our knowledge, not yet been transferred to mHealth apps, we subsequently integrate O'Neill's concepts into a relational concept of trust(worthiness) with four relata. Against this background, a case-based discussion of the introductory example of Kyle results in a conceptual analysis. Thereby, Kyle's situation illustrates the complexity of trustworthiness issues in the context of mHealth. The study of the situation focuses exemplarily on the avatar as the trustee and its task of providing knowledge. The analysis shows that O'Neill's account offers an encompassing account for reflecting the different dimensions and aspects of trustworthiness involved in using a mHealth app.

### Technological background: mobile apps for mental health

Mobile apps for treating mental health issues have significantly increased in their quantity in the last decades. They target a variety of disorders such as depression, anxiety, bipolar disorder, psychosis, post-traumatic stress disorders, substance use disorders, suicidal behaviors, and sleep disorders. Several reviews on the quality and effectiveness of mobile apps for mental health have already been conducted (Bakker et al. 2016; Grist et al. 2017; Lecomte et al. 2020; Wang et al. 2018). Nevertheless, there is still a lack of scientific evidence, randomized controlled trials, and meta-analyses to make clear recommendations concerning the use of apps for mental health issues.

Our analysis focuses on mobile apps, for example, installed on a smartphone or tablet, that can be classified as stand-alone self-management devices. Stand-alone means physicians or psychotherapists are not directly involved in the app use. Some apps are publicly available and can be purchased, for example, via app stores; others can be prescribed by a physician or psychotherapist. Consequently, not all users are necessarily patients and have been given a confirmed diagnosis. Although apps for mental health function differently, they generally record data (actively or passively) and try to positively influence the user's (mental) health behavior. In the following, mobile apps for sleep disorders with therapeutic intent will serve as a case study. Such apps are often based on CBT for insomnia. To reduce the users' insomnia symptoms, the users learn to establish sleep-promoting behaviors, optimize sleep times and replace sleep-disturbing inner attitudes with sleep-promoting ones.

The users can interact with the apps in different ways. Some of the apps use automated conversational agents (chatbots) or avatars. 'Chatbots are systems that are capable of conversing with users in natural language in a way that simulates the interaction with a real human' (Safi et al. 2020). Users can ask questions to which the system responds; or vice versa, the system asks questions the user is supposed to answer. In mental health, for example, Wysa is a chatbot that interacts with users to help with signs of anxiety and depression (Inkster et al. 2018). Chatbots usually lack human features, such as age or gender, so that users may perceive them as more unbiased, hence more neutral, and are more willing to share intimate medical information with them, for example, about sexually transmitted infections (Parviainen and Rantala 2022). The observation that some people are more willing to talk to a chatbot about intimate conditions than a ‘real’ doctor allows various interpretations. Shame, fear of being judged, and discretion can play significant roles. In addition, a link to trust can be established: If chatbots do not have human characteristics, they may be seen as more unbiased and more trustworthy because of their perceived neutrality. In addition, the trustworthiness of chatbots seems to be strongly influenced by their perceived expertise. People are more inclined to trust chatbots, for example, in customer service, if the chatbots display sufficient expertise (Nordheim et al. 2019). At the same time, as a lack of human presence characterizes automated chatbots, patients may lack trust in the abilities of chatbots, which can lead to concerns, for example, about accountability (Parviainen and Rantala 2022).

Avatars such as Alex from our introductory example can be understood as 'artificial computer-animated representations of humans within virtual environments' (Pan and Steed 2016). In contrast to chatbots, avatars can also provide non-verbal cues such as gestures, posture, movements, and facial expressions. Avatars can be used intentionally as a way of building trust, for example, through avatar-mediated communication between the users of a shared device or in a common virtual space (Bente et al. 2008; Steptoe et al. 2010; Junuzovic et al. 2012). Especially in the context of mental health conditions, such as sleep disorders, trust appears to be relevant to the efficacy of the therapy via the app (Gaebel et al. 2014). At the same time, the artificial nature of avatars can also lead to a loss of trust on the side of the users (Pan and Steed 2016).

### Autonomy, trust, and trustworthiness in the context of mHealth

Autonomy, trust, and trustworthiness are closely interlinked in bioethical debates. Several authors have examined the extent to which autonomy, trust, and trustworthiness are interrelated, especially regarding healthcare (O'Neill 2002; Oshana 2014; Nys 2015; Steinfath and Wiesemann 2016; McLeod and Ryman 2020; Myskja and Steinsbekk 2020). The discourse no longer only focuses on human individuals but integrates non-human systems such as robots or avatars and cooperation between humans and these systems (Noorman and Johnson 2014; Alaieri and Vellino 2016; de Visser et al. 2018). However, in the context of digital and mobile health technologies, many analyses focus on the individual user's (health-related) autonomy without conceptual cross-references to questions of trust and trustworthiness (Marijn et al. 2018; Owens and Cribb 2019; Schmietow and Marckmann 2019; Laacke et al. 2021). Despite their close connection, both concepts (autonomy and trust) tend to be discussed and conceptualized separately, only to be subsequently related to each other via the use of apps. The relevance and close connection of trust and autonomy in the context of (digital) mental health, however, suggests that there is also a profound conceptual connection. This calls for an approach that allows uncovering the conceptual links between autonomy and trust. Such an approach has been offered by O'Neill who notably takes this close relationship between trust and autonomy as a starting point for her account.

## Autonomy, trust, and trustworthiness according to O'Neill

In the following, we will introduce and further develop O'Neill's account of autonomy, trust, and trustworthiness because it allows us to link the concepts of autonomy and trust(worthiness) to one another. Since, in O'Neill's understanding, autonomy is the basis for relations of trust, we will, firstly, introduce O'Neill's concept of autonomy and, secondly, her conceptualization of trust and trustworthiness. O'Neill's approach will be used to shed light on the complexity of issues of trust(worthiness) in the context of mobile mental health. To transfer her account to mHealth apps, we will integrate O'Neill's concepts into a relational concept of trust(worthiness) with four relata. The example of Kyle described in the introduction will be used as a case study to explore whether and in what sense it can be said that Kyle legitimately trusts Alex, a virtual agent.

### Autonomy as universal self-legislation and prerequisite for trust

Current public and bioethical debates on digitization in healthcare commonly use individualistic concepts of autonomy. O'Neill criticizes such individualistic understandings of autonomy as inappropriate for medical practice, bioethical issues, and morality in general (Manson and O'Neill 2017) since they tend to reduce (patient) autonomy to the individual's right to be informed and to decide for or against an intervention. Instead, O'Neill builds upon Kant's concept of autonomy as self-legislation and develops an approach to principled autonomy (O'Neill 2013a). Based on Kant's idea that autonomy mainly consists in acting on certain forms of principles, namely principles that can be universalized, O'Neill understands autonomy as universal self-legislation (O'Neill 2002). Principled autonomy is expressed in actions whose principles could be adopted by all, and acting autonomously means acting in a morally qualified way (O'Neill 2002). In O'Neill's approach, autonomy as universal self-legislation refers neither to characteristics of the agents (for example, personal preferences or conceptions of a good life) nor to external aspects (for example, manipulation or pressure from third parties). Autonomy is not an attribute of individual persons, but the formal structure of principles that can serve all, respectively, could be law for all (O'Neill 2002). The point is not to find principles that everybody can act on at all times or places but to find principles that any agent can will as universal laws (O'Neill 2002).

O'Neill's account of principled autonomy founds the obligations to base our actions on universalizable principles and, second, to reject all principles that cannot be willed as principles for all (i.e., that are not universalizable). O'Neill emphasizes that by taking fundamental obligations (rather than rights) as a basis of her approach, the focus is shifted away from individualistic accounts and, instead, the 'relationships between obligation bearers and right holders, including institutionally defined relationships' (O'Neill 2002), come into the center of considerations. As we will elaborate in the next section, these fundamental obligations between obligation bearers and rights holders, grounded in the concept of principled autonomy, form the basis for relations of trust and trustworthiness. As part of her critique of individualistic concepts of autonomy, O'Neill stated that the importance of trust and trustworthiness had been underestimated in many debates. The conceptual and intrinsic link of autonomy and trust offers an opportunity to attribute to the role of trust the relevance that it should actually have.

What sort of obligations can be derived from the concept of principled autonomy? O'Neill's account does not provide a fixed list of obligations but rather a tool to analyze and answer this question on a case-by-case basis. Nevertheless, some general ethical requirements can be derived from the concept of principled autonomy, especially in the field of healthcare. Among these is, for example, the obligation to reject any forms of coercion and deception, which together provide the basis for relations of confidentiality and trust (O'Neill 2002). Coercion and deception cannot be willed as principles for all, as any attempt to will these as universal principles entails inherent contradictions. Universal coercion would mean that we would have to will others exert coercion on us, depriving us of the means to act and, hence, exercise coercion. This makes universal coercion a contradictory and impossible project (O'Neill 2002). A similar line of argumentation applies to the fundamental obligation to reject any form of deception, which provides the basis of relations of trust and trustworthiness. Deception cannot be willed as a principle for all, as this would undermine any relations of trust, which are a necessary precondition for any attempt to deceive. Universal deception is, therefore, in itself, a contradictory idea (O'Neill 2002). Through this structure of reasoning and the resulting conclusions, the principled autonomy approach provides a basis for free and informed consent, respecting persons and conceptions of confidentiality, and hence, provides reasons to establish and respect trustworthy relations. Autonomy, understood as universal self-legislation, is, therefore, the basis for relations of trust.

In contrast, individual(istic) concepts of autonomy often reduce autonomy to the individual's capacities and rights to decision-making, free choices, or avoidance of risks. Such conceptions of autonomy ignore dependence on others, aspects of recognition, and bilateral obligations that are commonly seen as prerequisites of trust. In O'Neill's account of principled autonomy, the mutual obligations found a universal net of obligations between moral agents. In this regard, O'Neill's account also goes beyond conceptions of relational autonomy that focus on social relationships, historical and socio-cultural circumstances, and relationships of (self-)trust as constitutive for or causally linked to autonomy. A distinctive feature of O'Neill's approach is that universal principles (and the respective obligations) are independent of the individuals affected, their social relationships and socio-cultural circumstances. Her account highlights that in issues of trust and autonomy we rely on a net of universal moral obligations that transcend certain circumstances at a specific time, and that this net of moral obligations needs to be considered to guarantee that trust will be properly placed. Even though individual(istic) and relational concepts of autonomy can be linked to concepts of trust, they generally do not provide intrinsic criteria to decide *how* to place trust appropriately. The advantage of O'Neill's approach is that it is informed and guided by 'practical reason'. In O'Neill's account, there is not only a conceptual link between autonomy and trust, but—as we will show—also the issue of well-placed trust.

### Trust and trustworthiness by O'Neill

In O'Neill's approach, trust is understood as the appropriate reaction to trustworthy agents. Trust is a reaction following a judgment of trustworthiness. For O'Neill, it is important that trust discriminates, meaning that it entails the judgment that persons are trustworthy in certain aspects and not others (O'Neill 2014). According to O'Neill's account, individuals should not be judged as trustworthy in general, but rather trustworthy regarding specific tasks and areas of competence. For example, a physician might be trustworthy regarding giving an injection but not for tasks outside medicine, such as repairing a car. Well-placed trust is based on the corresponding judgment that a person can be trusted to perform certain specific activities (O'Neill 2014).

Misplaced trust can have disastrous consequences. The ever-present risk of misplacing trust is, therefore, a compelling reason to find out how to place trust appropriately (O'Neill 2013b). In order to do so, we need to know whether the other party is trustworthy in performing a specific task. Consequently, an account of well-placed trust needs an account of trustworthiness. However, how can one properly judge how trustworthy someone is in certain aspects? O'Neill suggests three criteria for the judgment about trustworthiness: (1) honesty, (2) competence, and (3) reliability (O'Neill 2017). In deciding on trustworthiness issues, we have to judge '[…] about who will tell the truth, who will live up to their commitments, and who is competent at tasks they have taken on' (O'Neill 2017). O'Neill illustrates the three criteria with the aid of different examples: When one trusts the claims of a journalist, one trusts in the honesty and truthfulness of these claims, in this case, in their empirical truth. When trust is placed in a dentist to pull a tooth, one trusts their competence, namely their experience and skills. Moreover, if trust is placed in bank employees to send a monthly statement of account, trust is laid in the commitment of the bank employees to do so. Of course, in trusting our bank employee or dentist, we also assume that their statements are true and honest. However, the trust in their services is not only based on this belief but also assessments of their competence and reliability (O'Neill 2018). None of the three aspects (honesty, competence, or reliability) is sufficient in itself to build trust. However, depending on the specific situation, they are usually combined in different weightings in judgments of trustworthiness.

O'Neill draws a distinction between trusting the truth claims of others on the one hand and trusting their competencies and commitments on the other: Trusting the truth claims of others means judging whether or not these claims fit the world as it is. Trusting the competence or commitments of others means judging whether their actions meet the appropriate standards of competence and fulfill their commitments (O'Neill 2018). For O'Neill, the first type of judgment is empirical, and the second normative (O'Neill 2018).

Out of prudence, the other person's honesty, competence, and reliability should be assessed, not in general but concerning specific aspects and tasks (O'Neill 2013b). In O'Neill's understanding, placing or refusing trust is a matter of judging (O'Neill 2013b, 2014). Judgments can be used to correct prejudices or mistakes. We can change, confirm, or revise our judgments of trustworthiness. Thus, trust can be intelligently established by judgments. Placing trust intelligently requires judging available evidence (O'Neill 2014). Well-placed trust, however, does not demand to consider complete evidence but rather a sufficient amount of evidence (of the trustworthiness) in the relevant matters (O'Neill 2014). Which amount and sort of evidence can be regarded as sufficient cannot be answered in general terms but varies according to the matters at stake and individual preferences (e.g., how important the matter in question is to someone) or willingness to take risks.

## Transferring O'Neill's approach

Before we return to Kyle, who is asked to trust Alex, the avatar of the sleep app, we will apply the idea of principled autonomy and trust to the use of mental health apps.

### Principled autonomy as a moral account for mHealth app use

O'Neill's approach of principled autonomy gives an encompassing moral account for the sensitive situation of using apps for mental health. As principled autonomy is expressed in actions whose principles could be adopted by all, central moral obligations can be derived. A distinctive feature of O'Neill's approach is that universal principles and their respective obligations are not only directed to the individuals directly affected but as universal obligations to all persons concerned. For example, the argumentation that deception cannot be willed as a universal principle is valid not only from the app users' perspective but also from the perspective of developers, providers, and all other stakeholders involved. As O'Neill offers an approach of universal moral principles that addresses all moral agents, her account can be used to derive a net of moral obligations for all parties involved in the broad context of mHealth app use. For example, the obligation not to deceive entails the moral obligation of developers, producers, and providers not to engage in false advertising, manipulation, or data theft. Due to the large number of stakeholders involved in trustworthiness of mental health apps, such a universalistic approach seems more appropriate than the more commonly used approaches, which focus on individual relationships, obligations, and rights.

One might wonder why the stakeholders involved should care about such a universal moral account. Assuming, however, that app developers, producers, and providers, as well as the organizations behind them, are moral agents, they are the addressees of unconditional moral requirements, regardless of whether they have any further interests in the development of trustworthy health apps. O'Neill's approach provides thus a broad moral account to which app developers, producers, and providers can and should orient themselves when bringing technologies such as apps for mental health onto the market. From the perspective of O'Neill's approach, the users of mental health apps should be able to expect that the app developers, producers, and providers adhere to universalizable principles (e.g., non-deception, non-coercion) and, for example, would not lie to them. Moreover, these expectations, in turn, form a prerequisite for trusting relationships.

O'Neill's account emphasizes that issues of trust and trustworthiness in mHealth cannot be conceptualized as a particular relation between an app user and the provider, developer, or physician prescribing the app but rather as a net of universal obligations between all agents involved. For example, Kyle trusting their GP in prescribing the app entails believing that the GP themselves regards the app and the company behind it as trustworthy enough to prescribe it to patients suffering from sleep disturbances. As we will show in the following, this net of universal obligations represents an advantage over approaches conceptualized in terms of individual rights.

### Trust and trustworthiness in the situation of mHealth app use

The conclusion that the app users should be able to trust the app developers and all other agents involved represents a normative request; practically, the users are faced with the difficulty of deciding which providers or apps to trust. Potential app users might feel overwhelmed by too many app offerings and very complex regulations, for example, data privacy statements. In most cases, potential users cannot oversee and control everything, so they must invest a certain amount of trust. The question, then, is not whether the app users should give trust or not but how they can intelligently decide which apps they should trust. To discuss this issue more precisely, it is useful to break down the relationship of trust even further.

As relational concepts, trust and trustworthiness manifest in relations between different reference points, for example, between the app user and developer. The concepts range from a minimal understanding of trust as two-place relation (Domenicucci and Holton 2017) to concepts of trust with up to five (e.g., Castelfranchi and Falcone 2009) or even six relata (e.g., Baier 2013). Although different relational models of trust are discussed in the literature, a definition of a three-tier relationship is usually assumed: A trusts B to do Z. Likewise for trustworthiness: B is trustworthy to A regarding the performance of Z (Jones 2012, 2013).

**Fig. 1:**
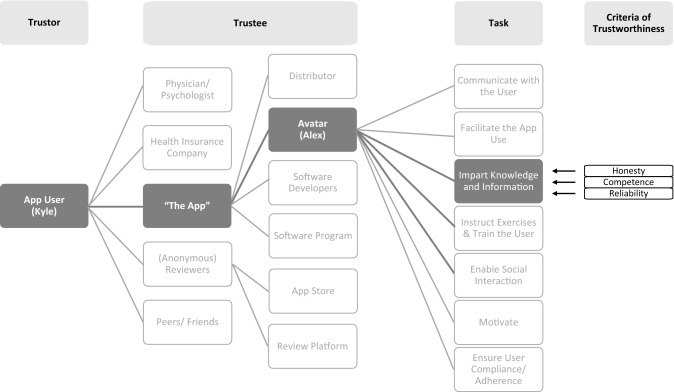
Relata and relation of trust(worthiness)

In O'Neill's approach, trust is understood as an appropriate reaction to trustworthy agents: a trustworthy agent B is given trust by A. Since a person should not be seen as trustworthy in general, but regarding specific tasks or areas of competence, the relation can consequently be formulated as: B is trustworthy to A regarding the specific performance of Z. In O'Neill's understanding, placing or refusing trust is a matter of intelligent judgment (O'Neill 2014). Given the importance of reasons why someone should be considered trustworthy in O'Neill's account, we suggest adding a fourth relatum to the three-tiers-model: B is trustworthy to A regarding the performance of Z *because of C*. This four-sided model of trustworthiness will now be applied to the introductory example of Kyle using the sleep app. Figure 1 illustrates the complexity that Kyle might face.

### Kyle's situation: a case-based discussion

Based on the four-tier model, the case study of Kyle's situation specifies who is trustworthy to whom for which activities and based on what criteria. It will become evident that the interpretation of trustworthiness in app use is multi-layered, and the model's relations are often interwoven. Hence, our case study has two limitations: We cannot provide a comprehensive picture and analysis of all relations and inter-relations. Instead, we will focus on links that are either unique to mHealth apps for mental health or have particular challenges. The second limitation is inherent to a case study: For each case study and analysis, the relata and relations involved have to be identified, and the relevance of the trustworthiness criteria may vary extensively in each case study. By referring to the complexity of assessing the trustworthiness of sleep and other health apps, we do not intend to imply that a single user should assess *all* of these aspects before trusting and using an app. In most cases, this is not even possible for an average user. Applying O'Neill's approach to mental health apps, however, illustrates how extensive the network of universal obligations is that users ultimately depend on when trying to assess the trustworthiness of apps.

In order to reduce complexity, we will focus on the app user (here: Kyle) as a trustor, i.e., the person who trusts. This reduction already represents a simplification, as the GP's trust in the app as a valuable tool to improve sleep quality certainly plays a significant role in our case study. It is further assumed that the app user is an individual adult person who uses the app for themselves. Although different users or user groups have different conditions, accesses, and abilities to use and evaluate health apps, we assume a non-disabled adult single person as app user to simplify the considerations for now. Children, youth, and other specifically vulnerable user groups or further relational aspects involved in sleep tracking (for example, between couples) are not dealt with.

In the quote cited in the introduction, Kyle is explicitly asked to trust Alex, the avatar. However, Alex is only one trustee out of many, and it might be that Kyle directs their trust not specifically to the avatar but to *the app*. On closer inspection, the app runs because of the work of different stakeholders such as software developers (as individuals), the company providing and distributing the app (as an organization), the computer software program (as a system), or the app store from which the app was downloaded. In addition, the relation of trust can refer to persons or institutions recommending the app, such as Kyle's GP, their health insurance company, Kyle's peers, or friends. Moreover, (anonymous) reviewers, for example, in the app store, can be recipients of trust. Given that scholarly literature regularly covers the topic of trust regarding companies or organizations (Wiencierz and Röttger 2016; Kramer and Tyler 1996), between patients and physicians (Illingworth 2010; Hawley 2015; Segers and Mertes 2022), and also users' trust in review platforms, electronic word-of-mouth, and electronic markets and corporations (Kang and Hustvedt 2014; Lee and Hong 2019; Grabner-Kraeuter 2002; Martínez-Navalón et al. 2021), we will focus on the relation between Kyle and the app. Moreover, as Kyle is explicitly asked to trust Alex, the following analysis focuses on the avatar. This seems especially worthwhile since Alex is a component of the app that shows up as an artificial agent with human features such as facial expressions, gestures, and speech. We will discuss the question of which task an avatar can be considered trustworthy. To do so, we use O'Neill's trustworthiness criteria, i.e., honesty, competence, and reliability.

The question cannot be answered straight-forward but requires mapping the tasks that the avatar is supposed to fulfill. This illustrates the complexity of the issue of trust, as different users might ascribe various tasks to an avatar and various degrees of importance to these tasks, such that succeeding or failing in these tasks can have a different impact on trust and trustworthiness issues. From an observational role, the tasks of Alex are manifold: He keeps in touch with Kyle, communicates with them, and facilitates the app use for them, for example, by guiding them through the sessions and being available in case of questions. Given that digital and mobile sleep apps require continuous user commitment to be effective, Alex may also take over the role as a motivator to ensure Kyle's adherence to the training provided and compliance with the advice given. For an app that is based on CBT, three further tasks can be ascribed to the avatar: imparting information and knowledge (the cognitive component, regularly conducted as psychoeducation), being a trainer and instructor for the mindfulness or breathing exercises that are meant to help the user to sleep better (the behavioral component), and enable social interaction with the user by para-verbal and non-verbal features (the therapeutic component in a narrow sense). In light of this extensive yet incomplete list of tasks, only the task of knowledge provision will be discussed in detail to give an idea of how to analyze trust issues using O'Neill's approach.

So far, three out of four relata of trustworthiness have been exemplified: Alex (B) could be seen as trustworthy to Kyle (A) regarding knowledge provision (Z). For the fourth relatum (because of C), O'Neill's trustworthiness criteria come into play and will help elaborate on why Alex is trustworthy or not (see Fig. 1). Following O'Neill's approach, Kyle has to look for reasonable evidence to place trust in Alex’s tasks and evaluate each task (here: knowledge provision) according to the criteria of honesty, competence, and reliability.

#### Knowledge provision

One of Alex's main tasks is to deliver knowledge about sleep and sleep disorders so that the app users can better understand and correctly assess their sleep problems. The information includes, for example, information on the sleep phases, the sleep rhythm, and the optimal amount of sleep. Basic, well-founded information can help correct misconceptions about sleep, decrease sleep-related maladaptive thoughts, and improve subjective sleep perception (e.g., Quintiliani et al. 2020). Suppose Kyle wants to check whether Alex is trustworthy regarding the task of knowledge provision. In that case, they have to look at this task from the three criteria of honesty, competence, and reliability. The criterion of honesty plays a significant role. When Kyle trusts Alex's claims, they foremost trust the truthfulness of these claims, for example, their empirical truth. Kyle can assess the truthfulness of Alex's claims by checking, for example, whether the sources of the information are made transparent and whether these sources are well-founded and scientifically validated. Additionally, Kyle could compare the information that Alex gives them with knowledge on sleep quality and sleep disturbances provided by other reputable sources. In O'Neill's account, well-placed trust requires consideration of a sufficient amount of evidence. However, O'Neill, unfortunately, does not give a general answer which amount and sort of evidence can be regarded as sufficient. This varies according to the matters at stake. In the case of Kyle, it depends on the individual situation and personal resources that Kyle has and wants to use. Kyle can decide for themselves how much and which evidence they classify as sufficient. According to O'Neill's account, the company developing the app has the obligation to make it as easy as possible for Kyle to check the truthfulness and, hence, trustworthiness of the information that Alex gives.

The assessment of competence and reliability takes a very similar form in the task of knowledge provision. Alex presents himself at the beginning of the app as a sleep expert and promises that all the advice he gives is scientifically based (Somnio). Kyle can verify the validity of these promises by checking to what extent sleep therapists and specialists were involved in the app's development, whether the sources cited by the app provider are up to date and whether the information is regularly updated. Of course, not every piece of information can and should be checked by Kyle. If everything is controlled, there is no need and place for trust. Nevertheless, it is very difficult to decide how much checking is prudent enough. In any case, the developing company has an obligation to make it as easy as possible for Kyle to access this information. By providing these references, Kyle is given some indication of the trustworthiness concerning the task of knowledge provision. Kyle receives further indications of competence and reliability by observing whether the avatar offers them helpful information at the right time and in the event of questions that Kyle directs to the avatar. The more requirements are met, the more legitimized it is to trust Alex for the task of providing knowledge. However, there is no general threshold for trustworthiness and well-placed trust. Instead, this threshold varies according to the matters at stake (e.g., the severity of the sleep disturbances and the associated degree of vulnerability of the user) and individual preferences regarding the trustworthiness of third parties.

Part of these considerations can also be transferred to the other two tasks of CBT, namely the behavioral and therapeutic components. Here again, Kyle can verify the validity of Alex's promises by checking to what extent sleep therapists and specialists were involved in the app's development, whether the sources cited by the app provider are up to date and whether the effectiveness of the app has been tested and proved in appropriate, randomized and controlled clinical studies. Kyle receives further indicators of the competence and reliability of the avatar in accomplishing the behavioral training by following the mindfulness or breathing exercises and documenting in a sleep diary whether they actually improve their sleep quality. Kyle can gather information about the avatar's trustworthiness in performing the therapeutic task by relying on verified user ratings and comments and testing the avatar with unexpected questions and reactions to see how the artificial agent reacts. It may well turn out that the trustworthiness of the avatar varies with regard to these different tasks of CBT, e.g., that Kyle judges Alex as a trustworthy knowledge provider and 'tool' for psychoeducation, but (at least at the current state of AI development) as insufficient with regard to the therapeutic and social component of CBT.

#### Analyzing the complex interrelations of trust and trustworthiness in mental health apps

In the context of app use, the app developers and the distributing organizations are 'faceless'. As the app can simply be downloaded from the app store, there is no human contact and no face-to-face relationship. This differs from therapeutic relationships between a patient and a physician or therapist, in which the potential trustee is more easily identifiable for the person suffering from sleep disturbances. However, the avatar in the app gives it a face. This might affect (trust) relations as people often attribute human characteristics to apps, for example, chatbots, avatars, or AI (Ryan 2020). Whether the fact that people associate human activities and skills with AI-based systems leads to more (or less) trust depends on various aspects and requires more empirical research. However, that Alex can be judged as trustworthy in performing certain tasks does not necessarily mean that Kyle mistakenly anthropomorphizes the avatar. Kyle may be fully aware that Alex actually is not a sleep therapist, that he did not research all the scientific knowledge on sleep and sleep disturbances on which his task of knowledge provision is based, but that this was done by sleep experts and therapists involved in the development of the app. Nevertheless, O'Neill's account helps to clarify that placing trust in an app cannot be conceptualized as a particular and individual relation between an app user and a provider, developer, physician prescribing the app, or an artificial agent. Instead, by transferring O'Neill's account to the field of mental health apps, it becomes clear that issues of trust involve a net of universal obligations between different agents involved in the app development, provision, and maintenance, that users ultimately rely on when trusting an app. This net of universal obligations is a *prerequisite* for putting trust in individual tasks that an app, or avatar, is supposed to fulfill. At the same time, O'Neill's account offers a tool to structure these complex situations.

Our analysis suggests that based on O'Neill's account, judgments of trust and trustworthiness in using health apps involve a set of universal obligations that address all the agents involved. These obligations—as universal obligations—cannot be diverted and form the necessary background for the trust a user invests in using a specific app. This refutes the criticism that placing trust in AI is actually misplaced trust, as all an avatar or AI can deliver is reliability. Of course, the avatar itself is not an autonomous person or a bearer of obligations. However, by relying on the universal obligations of all the persons involved in the development, maintenance, and recommendation of mental health apps, users can prudently and intelligently assess the trustworthiness of the avatar in accomplishing the tasks it is designed to fulfil. O'Neill herself gives the example of the trustworthiness of institutions in fulfilling certain tasks (e.g., we trust our bank in sending monthly statements of account, O'Neill 2018), even though institutions, too, cannot be regarded as moral and autonomous persons in a strong Kantian sense. Although O'Neill argues in part of her work in favour of a 'thin concept of agency' that allows us to consider some institutions and artificial agents as capable of agency in the sense of an ability to '*integrate* capacities to *reason* and to *act*, and to maintain some *independence* from other forces and agents' (O'Neill 1986), our argumentation does not rely on the assumption that the avatar (or the app) should be considered as an agent in the literal sense. It is sufficient to view the avatar as an extension of the trustworthiness of the collective effort and universal obligations of all the persons involved in the app's development, maintenance, and implementation. With regard to communication technology, O'Neill highlights the fundamental role of such intermediaries. For her, intermediaries might be 'institutions and office-holders; others are components or aspects of communication systems and internal institutional processes including algorithmic processes' (O’Neill 2020). For the latter cases, namely algorithms that create or alter the content (such as the avatar Alex), O'Neill points to (institutional) culture and formal law as they 'can then supplement the evidently incomplete approach to judging trustworthiness that […] digital processes offer' (O'Neill 2020). By considering, e.g., the developing institution's culture and relying on the universal net of obligations, users are enabled to prudently judge the trustworthiness of an avatar in performing specific tasks. Furthermore, O'Neill's approach also brings a new perspective to the allegation that this form of 'misplaced trust' in chatbots, avatars, or apps diverts responsibility from developers, providers, and people using them (Ryan 2020). On the contrary, especially in apps using CBT, an avatar can be helpful in the implementation of the training program and have beneficial effects on the effectiveness of therapy without (as we suggested) necessarily diffusing obligations.

## Conclusion

O'Neill's approach of autonomy, trust, and trustworthiness shows that it is important not to speak of trust in general but to specify who is trusted, concerning which tasks, and according to which criteria. This specification can provide orientation in complex situations characterized by questions of trust and help to place trust intelligently. The example of sleep apps has revealed complex relations of trust between trustor, potential trustees, multiple tasks, and trustworthiness criteria. Since the relations are manifold, an individual assessment must be made by the user of each application and its specific tasks. O'Neill's approach, complemented by the four relata of trust, is instructive to systematize how, for whom, and in which regard trust and trustworthiness play out in the domain of mHealth applications for mental health.

For a thorough assessment, the crucial point is well-placed trust. O'Neill provides the normative criteria to judge the trustworthiness of third parties for certain tasks. However, it must also be possible for the users to apply these criteria practically and distinguish between trustworthy and untrustworthy apps. For this, the user's ability to differentiate between well-placed and misplaced trust in mHealth apps must be strengthened. App developers can and should use the three criteria of trustworthiness as a guide to structure and provide the relevant information that users need to assess trustworthiness and build trust in using an app. Of course, reliably assessing the trustworthiness of health apps also requires a certain amount of digital and health literacy on the side of the app user. Different users or user groups have different resources, accesses, and abilities to check the trustworthiness of apps. Due to their obligation to enable users to assess the trustworthiness of their services, the developer companies must address the different resources and diverse requirements of the user groups that serve as the app's target group. How this can be achieved in practice is also, in part, an empirical question, that should be at the center of further research. Moreover, the conceptual and normative considerations outlined above should be applied to the developers and the other stakeholders involved. The comprehensive picture of mHealth arising out of such efforts will help oversee the complexity and interwovenness of (digital) healthcare and further policy endeavours in the field of 'trustworthy AI'.

## Data Availability

All data analysed and that support the findings of this study are included in this published article.

## Abbreviations

App: Application
AI: Artificial intelligence
CBT: Cognitive behavioral therapy
GP: General practitioner
mHealth: Mobile health

## Footnotes

1 They, their, them are used in the examples as gender-neutral pronouns.

2 CBT is a form of psychological intervention that is effective for various problems and mental illnesses such as depression. CBT interventions usually involve changing thinking and behavioral patterns (American Psychological Association 2017).

3 This sequence is based on 'Somnio', a health app developed to treat and support patients suffering from sleep disturbances. Somnio has been approved by the German Federal Institute for Drugs and Medical Devices. This means that physicians and psychotherapists can prescribe the app and German health insurance companies pay for the costs of the app. For further information on the app, see https://somn.io/en/ (last checked: 17 Jan 2023).
